# Replication of Alphaviruses: A Review on the Entry Process of Alphaviruses into Cells

**DOI:** 10.1155/2011/249640

**Published:** 2011-07-02

**Authors:** Jason Yat-Sing Leung, Mary Mah-Lee Ng, Justin Jang Hann Chu

**Affiliations:** Laboratory of Molecular RNA Virology and Antiviral Strategies, Department of Microbiology, Yong Loo Lin School of Medicine, National University Health System, 5 Science Drive 2, National University of Singapore, Singapore 117597

## Abstract

Alphaviruses are small, enveloped viruses, ~70 nm in diameter, containing a single-stranded, positive-sense, RNA genome. Viruses belonging to this genus are predominantly arthropod-borne viruses, known to cause disease in humans. Their potential threat to human health was most recently exemplified by the 2005 Chikungunya virus outbreak in La Reunion, highlighting the necessity to understand events in the life-cycle of these medically important human pathogens. The replication and propagation of viruses is dependent on entry into permissive cells. Viral entry is initiated by attachment of virions to cells, leading to internalization, and uncoating to release genetic material for replication and propagation. Studies on alphaviruses have revealed entry via a receptor-mediated, endocytic pathway. In this paper, the different stages of alphavirus entry are examined, with examples from Semliki Forest virus, Sindbis virus, Chikungunya virus, and Venezuelan equine encephalitis virus described.

## 1. Alphaviruses

Alphaviruses are primarily arthropod-borne viruses (arboviruses) within the family *Togaviridae*. Viruses belonging to this genus are often classified as either New World alphaviruses, or Old World alphaviruses, depending on the geographic location from which they were originally isolated [1]. New World alphaviruses, including Eastern equine encephalitis virus (EEEV), Venezuelan equine encephalitis virus (VEEV) and Western equine encephalitis virus (WEEV), typically cause encephalitis in humans and other mammals, whereas Old World alphaviruses, such as Chikungunya virus (CHIKV), O'Nyong-Nyong virus (ONNV), Ross River virus (RRV), Semliki Forest virus (SFV), and Sindbis virus (SINV), cause a fever, rash, and arthralgia syndrome that rarely causes fatality [2]. While early studies on alphaviruses focused on the prototypic SFV and SINV due to their ability to grow to high titres in cell culture while being nonpathogenic to humans, recent attention has been directed towards investigating CHIKV. The 2005 outbreak of CHIKV in La Reunion infected 40% of the 785,000 population, resulting in 250 fatal cases [3]. The reemergence of CHIKV reiterates the potential threat that alphaviruses pose to human health, and the necessity to understand mechanisms involved in alphavirus biology.

## 2. Genomic Composition and Virion Structure

Alphaviruses are small, icosahedral-shaped, enveloped viruses, approximately 70 nm in diameter [4–6]. The alphavirus virion has a host-cell acquired lipid membrane [6–9]. Embedded within this membrane are 80 spikes, arranged in a *T* = 4 icosahedral [6, 9]. The glycoproteins E1 and E2 associate as heterodimer subunits, which are in turn assembled into trimers to form the spike protrusions [9–11]. Both E1 and E2 are transmembrane proteins with C-terminal cytoplasmic regions that are thought to interact with the nucleocapsid [12, 13].

**Figure 1:**
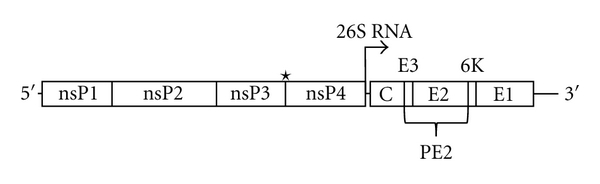
*Alphavirus genome.* The alphavirus genome is single-stranded, positive-sense RNA, encoding two open reading frames. The nonstructural proteins are translated from the genomic RNA while the structural proteins are translated from subgenomic 26S RNA (promoter as indicated). The two polyproteins are cleaved by viral cysteine, and host proteases to generate the individual protein products. *denotes leaky stop codon.

The alphavirus genome is a single-stranded, positive-sense, RNA genome approximately 12 Kb in length [14, 15]. In addition to genomic length RNA, subgenomic RNA encoding the structural proteins is also generated, with both species containing a 5′ cap and a poly(A) tail [14–16]. The coding sequence consists of two large open reading frames (ORFs); the N-terminal ORF encodes the nonstructural polyprotein while the C-terminal ORF encodes the structural polyprotein (Figure 1). The two polyproteins are cleaved posttranslationally by viral (cysteine) and host proteases. The four nonstructural proteins (nsP1 to 4) and their cleavage intermediates are involved in RNA replication, with the five structural proteins (C, E3, E2, 6K, E1) and their cleavage intermediates required for viral encapsidation and budding (Figure 1) [15, 17, 18].

The alphavirus nsP1 possesses both guanine-7-methyltransferase and guanylyl transferase activities required for capping and methylation of newly synthesized viral genomic and subgenomic RNAs [19, 20]. During RNA replication, nsP1 is thought to anchor replication complexes to cellular membranes [21]. The alphavirus nsP2 exhibits RNA triphosphatase/nucleoside triphosphatase, as well as helicase activity within the N-terminal half [22–24] while the C-terminal half encodes the viral (papain-like) cysteine protease required for processing of the nonstructural polyprotein [17, 25]. Crystal structures of the CHIKV and VEEV nsP3 N-terminus indicate ADP-ribose 1-phosphate phosphatase and RNA-binding activity [26] while mutagenesis studies also reveal a role for nsP3 in modulating pathogenicity in mice [27, 28]. The nsP4 protein functions as the RNA-dependent RNA-polymerase (RdRp), containing the catalytic *GDD* motif in the C-terminus [29]. It has also been hypothesized that nsP4 acts as a scaffold for interaction with other nsPs or host proteins via its N-terminal [30], with adenylyl transferase activity also observed [31].

During nucleocapsid formation, the alphavirus capsid protein (C) binds viral genomic RNA via N-terminal Arg, Lys, and Pro residues [32, 33]. Mutagenesis studies identified a leucine zipper located within this region essential for formation of nucleocapsid-like particles, presumably mediating dimerization during virus assembly [34]. The protein C-terminal is the serine-protease domain [18, 35], which also contains a hydrophobic pocket for glycoprotein binding adjacent to the substrate-binding site [12]. The role of the structural protein E3 is currently undefined, and appears to vary between different alphaviruses. While the E3 protein of SFV is found associated with virions [36], the E3 protein is not incorporated into virions of other alphaviruses including CHIKV, SINV, or WEEV [37]. The E2 glycoprotein of alphaviruses responsible for receptor binding is embedded within the membrane courtesy of 30 C-terminal residues [38–40]. Amino acid changes identified the E2 protein as a determinant of neurovirulence [41–43]. Site-directed mutagenesis identified an Tyr-X-Leu tripeptide within the endodomain required for interaction with the capsid protease domain [12, 13, 44], in concert with conserved Cys residues that are modified by palmitoylation [45]. 6K is a palmitoylated structural protein essential for alphavirus particle assembly [46, 47], where it is thought to influence transport to sites of virion assembly at the plasma membrane, before being incorporated into virions in small amounts [46, 48, 49]. The alphavirus 6K protein has also been classified as a viroporin due to its ability to form cation-selective ion channels and alter membrane permeability in bacterial and mammalian cells [50–52]. The E1 protein is the alphavirus fusion protein [53, 54], with a fusion peptide residing within a highly conserved hydrophobic domain [38]. 

## 3. Alphavirus Life-Cycle

**Figure 2:**
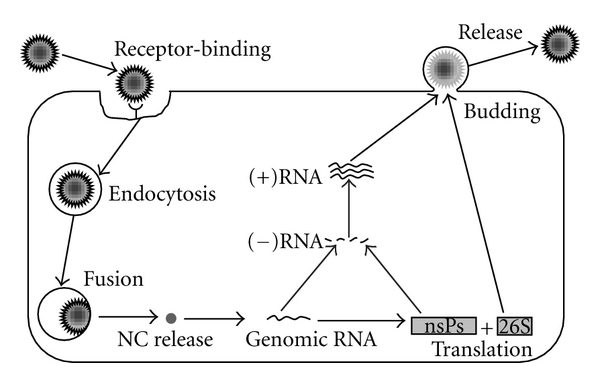
*Alphavirus life-cycle.* Alphavirus entry into cells is initiated by receptor-binding, followed by clathrin-mediated endocytosis. Fusion to endosomal membranes transports nucleocapsid (NC) into the cytoplasm, where RNA is released after disassembly. Genomic RNA is used for both translation of proteins from genomic and subgenomic (26S) RNA, and transcription of nascent (+)RNA via a (−)RNA template. The structural proteins translated from 26S RNA encapsidate nascent genomic RNA before budding from cells, and eventual release.

Upon entry, alphavirus particles undergo disassembly, releasing genomic RNA into the cytoplasm of infected cells (Figure 2). The viral genome is then translated from two ORFs to generate the nonstructural (P1234) and structural polyproteins [55]. Early in infection P1234 is cleaved in *cis* between nsP3 and nsP4 to yield P123 and nsP4 [56, 57]. P123 and nsP4 form an unstable initial replication complex, which is able to synthesize negative-strand RNA [1, 58–60]. Cleavage of P123 to nsP1 and P23 can only occur in *trans*, and only at a sufficiently high concentration of the polyprotein. The polyprotein products nsP1, P23, and nsP4 form a replication complex within virus-induced cytopathic vacuoles (CPV I) that are active in negative-strand synthesis, as well as genomic RNA synthesis, but not in subgenomic RNA synthesis [60–64]. After complete cleavage to nsP1, nsP2, nsP3, and nsP4, negative-strand synthesis is inactivated and the now stable replication complex switches to synthesis of positive-strand genomic and subgenomic RNA [58, 59]. In most alphaviruses, a leaky termination codon is present following nsP3 (indicated in Figure 2), with read-through estimated to occur with only 10–20% efficiency [65]. This leads to an excess of P123 nonstructural polyprotein compared to P1234, and a depletion of nsP4 relative to the other nsPs. Further diminishing intracellular nsP4 is a destabilizing tyrosine residue at the N-terminal which signals rapid degradation by the N-end rule pathway [66]. It is worth noting that expression of a small fraction of nsP4 in cells is relatively stable, presumably in the form of replication complexes, suggesting nsP4 is only degraded when in excess [66]. Removal of the destabilizing Tyr residue leads to poor RNA replication [67]. 

Cleavage of the structural polyprotein occurs cotranslationally, beginning with the autoproteolytic cleavage of the capsid protein from the remainder of the polyprotein [18, 68, 69]. C protein is then available to associate with newly synthesized RNA, recognizing specific packaging signals in the 5′ half of the genome, such that only full-length genomic RNA is packaged into nucleocapsid-like particles [32, 33]. The E3 protein acts as a signal sequence for insertion of the remaining polyprotein into the endoplasmic reticulum, where it is processed by host signal peptidase [18, 70]. Similarly, the 6K protein acts as a signal sequence for the downstream processing of the E1 protein [51].

Upon synthesis, the E2 glycoprotein precursor, PE2 (p62 in SFV), and E1 glycoproteins interact with each other (preferentially in *cis*) to form heterodimers [71–73]. These heterodimer complexes are then transported from the endoplasmic reticulum to the cell surface via the Golgi complex [74–76]. At a late stage of transport, the PE2 precursor is cleaved in its lumenal domain by host furin-like protease to generate mature E2 and E3 proteins [74, 76, 77]. This cleavage induces a conformational change that weakens the E1-E2 interaction in the spike heterodimer [11], priming the fusion peptide for activation upon exposure to low pH [78]. Interactions between the C protein and the cytoplasmic domain of the E2 protein drive the budding process, with E1-E2 heterodimers forming an envelope around nucleocapsid-like particles [12, 79, 80]. Upon release from cells, virions acquire a membrane bilayer derived from the host cell plasma membrane [6–9]. 

## 4. Receptor-Mediated Endocytosis

Viruses enter cells at the plasma membrane, either by fusion with membrane components at the cell surface, or by receptor attachment and internalization, followed by fusion with intracellular membranes of endocytic vesicles. Receptor-mediated endocytosis is the predominant mode of entry, most often mediated by the formation of clathrin-coated pits, and the subsequent transport to early endosomes, where the low-pH environment triggers fusion [81]. Alternatively, some viruses utilise clathrin-independent pathways to gain entry into cells. The caveolar/raft pathway transports internalised virus to neutral-pH caveosomes, before redistribution to the ER. There are also a number of clathrin-independent, caveolin-independent pathways that viruses use for cellular entry that rely on small GTPases, although these are not well understood [81].

The entry of alphaviruses into cells is facilitated by interaction of the spike E2 component with protein receptors on the surface of target cells (Figure 2) [40, 82]. A 63 KDa protein on the surface of avian cells was the first alphavirus receptor observed, although its identity was not determined [83]. Antibodies generated against BHK membrane proteins were screened to identify the 67 KDa laminin receptor as a high-affinity attachment receptor for SINV infection in mammalian cells [84]. Subsequent binding experiments of SINV to dendritic cells was shown to be SIGN dependent, with DC-SIGN and L-SIGN acting as receptor molecules [85]. Heparan sulfate, a cell surface glycosaminoglycan, may also act as an attachment receptor for alphaviruses [86–88]. However, the affinity of alphavirus binding to heparan sulfate seems to be acquired after serial passaging in cell culture, with field isolates displaying much lower affinity for heparan sulfate than laboratory-adapted strains [86–88]. 

Upon attachment to cellular receptors, alphaviruses are rapidly internalized and delivered to endosomes (Figure 2) [89–91]. The formation of clathrin-coated vesicles requires dynamin, a ~100 KDa protein that facilitates the budding of clathrin-coated pits, leading to the formation of coated vesicles, in a GTP-dependent manner [92]. When dominant-negative dynamin mutants specifically blocking the formation of clathrin-coated pits and vesicles were expressed [93, 94], entry of the alphaviruses CHIKV, SFV, and SINV were prevented [89, 95]. Similarly, a dominant-negative mutant of Eps15, another mediator of clathrin-dependent endocytosis, prevents entry of VEEV into cells [96]. Investigations performed using dominant-negative mutant forms of Rab5 and Rab7, genes important in endocytic trafficking to the early and late endosomes, respectively [97, 98], indicate that both SFV and VEEV are transported to early endosomes whereas only VEEV is transported to late endosomes, before fusion with target membranes [96, 99].

While the entry of alphaviruses is widely accepted to be dependent on clathrin-mediated endocytosis and fusion with endosomal membranes, the ability of alphaviruses to enter host cells via alternative mechanisms has also been reported. Supporting this hypothesis is an early study on SINV entry that showed the translation of viral RNA in the cytosol of cells, even when infected cells were treated with the weak bases chloroquine and ammonium chloride, suggesting infection involving acidic endosomes can be circumvented [100, 101]. More recently, the infection of various cell lines by SINV was shown to proceed in the absence of low-pH-induced endocytosis, indicative of entry via a clathrin-independent pathway [102–104]. Similarly, SFV infection of BHK and CHO cells following either normal virus fusion in endosomes, or experimentally induced fusion at the cell surface, highlighted the ability of alphaviruses to infect cells by an alternative pathway. Although CHO cells could only be infected following the endocytic pathway, BHK cells were able to be infected efficiently following fusion in either endosomes or at the plasma membrane, as evidenced by viral RNA and protein synthesis [105]. In agreement with this was the finding that SFV could be found inside noncoated pits and vesicles [106]. When siRNA was used to knock-down clathrin heavy chain, CHIKV infection of both HEK293 and HeLa cells was unaffected [107]. Interestingly, experiments using anti-clathrin antibodies showed only a ~60% block in SFV infection [90]. This partial block in infection could mean that either the antibodies do not inhibit the clathrin pathway completely, or that SFV can also enter through an alternative pathway that does not require clathrin. Such a scenario may also be true for CHIKV, where dominant-negative mutants of Eps15, Rab5, and drug inhibitors of endocytosis, showed only partial block in infection, supporting the hypothesis that several pathways are hijacked by CHIKV to penetrate into target cells [107]. 

## 5. Fusion

Early studies revealed the E1 protein of alphaviruses to be the fusion protein [38, 53, 54, 108–110]. Furthermore, removal of the E2 protein by protease digestion suggested that the E1 protein alone is sufficient for membrane fusion to occur [111]. However, fusogenic activity of the E1 protein is suppressed by interaction with the E2 protein [11]. Within endosomal vesicles, the E1-E2 heterodimer undergoes irreversible conformational changes upon exposure to pH of ~6 or below [91, 109, 112–115]. This low-pH environment liberates the E1 subunit from association with the E2 subunit, allowing the rearrangement to a homotrimeric complex active for fusion [108, 109, 116, 117]. E1 homotrimers associate with the target membrane via membrane insertion of the hydrophobic fusion peptide to form pores in both cellular and viral membranes for release of nucleocapsid into the cytoplasm (Figure 2) [111, 118, 119]. The fusion process occurs very rapidly, before alphaviruses are at risk of lysosomal degradation [115]. Treatment of cells with lysosomotropic weak bases chloroquine, concanamycin, ammonium chloride, bafilomycin, or monensin neutralizes the pH in endosomes, preventing fusion with membranes [91, 96, 99, 107, 120, 121].

In addition to a dependence on low pH, the fusion of alphaviruses to membranes requires the presence of cholesterol [107, 108, 114, 115, 122–125]. Small amounts of sphingolipid are also required in target membranes for an as yet unidentified role during the fusion reaction itself [114, 123, 126]. Cholesterol appears to be necessary for the hydrophobic interaction of the alphavirus E1 ectodomain with the target membrane leading up to fusion [113, 127]. However, this cholesterol-dependence differs amongst the alphaviruses; while the entry of CHIKV, SFV, and SINV is inhibited by the depletion of cholesterol, VEEV is still able to enter cells under similar conditions [96, 107]. It was proposed that variations between the envelope proteins of VEEV compared to other alphaviruses may account for these observed differences [125]. As predicted, cholesterol dependence of SFV and SINV were attributed to a specific residue within the E1 protein at position 226 [125]. Viruses containing an E1-P226S mutation are more efficient at fusion in the absence of cholesterol compared to wild-type, with the E1 protein converting to the active fusion homotrimer more readily [128, 129]. Sequence analysis of the VEEV E1 protein shows that this mutation is already present [96]. It has been shown that the membranes of early endosomes are enriched in cholesterol whereas late endosomes are depleted of membrane cholesterol [130, 131]. This has been used to explain the differences in cholesterol requirement, since SFV appears to undergo fusion in early endosomes while VEEV traffics to late endosomes, for fusion [96]. 

## 6. Nucleocapsid Disassembly

Due to steric hindrance, only a small fraction of the E1 fusion protein molecules present on the surface of an individual virus particle would be able to participate in fusion reactions within the endosomal membrane [132]. It has been proposed that the remaining fusion proteins that have not reacted with the target membrane may fold back and react with the viral membrane in which they are anchored, leading to the formation of ion-permeable pores [132]. Together, these processes allow the delivery of nucleocapsid into the cytoplasm (Figure 2), and a flow of ions through the target membrane. 

The formation of ion-permeable pores by viral proteins during entry has been found with other viruses such as influenza virus, with the M2 protein implicated in the flow of protons from the endosome at low pH [133]. A similar function for the alphavirus E1 protein has been proposed [111, 119]. Indeed, the accumulation of viral structural proteins in the cell membrane during virus multiplication alters the permeability of the membrane at low pH late in infection [118, 134]. When membrane permeability of cells incubated with SINV and SFV at low pH was assessed, voltage measurements confirmed the formation of ion-permeable pores [135]. The resulting flow of protons from the endosome into the cytoplasm through this pore would lead to a region of low pH, consistent with the discovery that a low-pH environment strongly stimulates disassembly of alphavirus nucleocapsid [136]. 

Fusion of the viral envelope with the endosomal membrane releases nucleocapsid into the cell cytoplasm (Figure 2). Uncoating of alphavirus nucleocapsids occurs almost immediately (~1 minute) after their penetration into the cytoplasm [137]. 60S ribosomal RNA interacts with the C protein, facilitating uncoating of the nucleocapsid and release of viral RNA for initiation of protein synthesis (Figure 2) [55, 137–139]. 

## 7. Conclusion

The study of alphaviruses has provided a number of insights relating to virus entry. In fact, reports on SFV were the first to demonstrate entry of viruses via receptor-mediated endocytosis, and the use of clathrin-coated vesicles [91]. The entry pathway of alphaviruses has been further elucidated, providing a greater understanding of events such as virus trafficking, fusion, and the release of genomic material into cells. However, there remain a number of questions that are yet to be resolved. One area for investigation is the identification of receptors used in alphavirus attachment to cells. So far, receptors have only been identified for SFV and SINV entry into cells. Isolating receptors could lead to the development of novel antivirals targeting alphavirus entry. Another topic for exploration is the use of alternative pathways for alphavirus entry into cells. There is growing evidence that alphaviruses are able to infect cells independent of clathrin-mediated endocytosis, either by employing a different entry pathway altogether, or being able to enter cells using several pathways. In the case of CHIKV, entry has also been shown to occur in the absence of cav-1 (required for caveolar vesicle formation) [140]. It may be possible that alphaviruses such as CHIKV are able to utilize a dynamin-dependent pathway reliant on the small GTPase RhoA [141]. This pathway does not involve clathrin, caveolae, or Eps15, yet is strongly inhibited by knock-out of dynamin, RhoA, or the depletion of cholesterol or sphingolipids, concurring with previous work. It is hoped that the continued study of alphaviruses will shed new light on the processes involved in entry.
